# N-Back auditory test performancein normal individuals

**DOI:** 10.1590/S1980-57642009DN30200008

**Published:** 2009

**Authors:** Vanessa Tomé Gonçalves, Letícia Lessa Mansur

**Affiliations:** 1 Speech pathologist, PhD in Neurogeriatrics. Laboratory of Neurolinguistics. Department of Physiotherapy, Speech Therapy and Occupational Therapy of the University of São Paulo School of Medicine, São Paulo SP, Brazil.

**Keywords:** age, working memory, cognition

## Abstract

The working memory construct refers to the capacity to maintain information for a limited time.

**Objectives:**

To devise stimuli and adapt the 5-back test and to verify the effect of age in normal Brazilian individuals.

**Methods:**

31 healthy adults (15 young adults and 16 older adults) were evaluated by batteries of auditory stimuli to verify the inter-group differences (age effect) in working memory span, total correct answers and intrusions, and the intra-group effect of type of stimulus.

**Results:**

There was no intra-group stimulus effect. Individuals from both groups processed di and tri-syllables similarly. No difference between groups (no age effect) was observed for any N-Back parameters: total score, span, number of intrusions, in either di or tri-syllable presentation.

**Conclusion:**

the processing capacity of 5 elements in phonological working memory was not affected by age.

## Abstract (pt)

Memória operacional é um construto que se refere à capacidade de manter a informação acessível para uso por tempo limitado.

**Objetivo:**

Elaborar estímulos e adaptar o teste N-Back auditivo e verificar o efeito de idade em indivíduos brasileiros normais.

**Método:**

31 sujeitos adultos (15 adultos jovens e 16 idosos) foram avaliados por baterias de estímulos auditivos para verificar diferenças inter-grupos (efeito de idade) na capacidade da memória operacional, número de respostas corretas e intrusões e também examinar o efeito do tipo de estímulo intra-grupos.

**Resultados:**

Não houve efeito significante do estímulo intra-grupo. Indivíduos de ambos os grupos processaram de modo similar di e trissílabos. Não houve diferença entre os grupos (efeito de idade) nos parâmetros: escore total, capacidade de memória operacional, número de intrusões tanto na apresentação com dissílabos como trissílabos.

**Conclusão:**

A capacidade de processar cinco elementos na memória operacional fonológica não foi afetada pela idade.

“Working memory (WM) is assumed to be a temporary storage system under attentional control that underpins our capacity for complex thought” (p.1).^1^ The Baddeley and Hitch WM model is based on three components: a main component, central executive and two subsystems: the phonological loop which deals with verbal material (sequences), and the visual sketchpad that handles visuospatial information. The central executive is responsible for relating information from support subsystems”.^1^ Variations may occur in any WM component. However, studies in healthy adults indicate an association between cognitive abilities and the executive attention component of WM. There is substantial evidence of decline in studies on normal older adults.^2,3^ Other sources of variation in WM include pathological conditions such as thalamic lesions,^4^ aphasia,^5^ dementia^6^ and traumatic brain injury.^7^

There are many approaches to WM, most of which have common features. An interesting proposal supports the specialized WM system for language, arguing that language needs WM applied to the construction of all levels – segmental and lexical phonological representations, morphology, intonational structure, syntax and discourse.^8^

## N-Back

N-Back is a frequently used instrument to measure WM. This test requires codification, temporary storage and response, as it is necessary for the individual to update and maintain information continuously in the WM to readily access it. The stimuli, usually 2 or 3 back-digits, which are either visual (N-Back visual) or auditory (N-Back auditory) presentations.^5^ Variants of N-Back have been proposed and include presentation of one or two syllable digits or objects.^4^ The measures of performance include reaction time, availability of target item, interference and decay or facilitation, increase in memory load and switch costs.^9,10^

The participant is instructed to answer when the current item is the same as that which was back presented, varying the position in each series.

This test is valuable because it does not solicit a verbal response and thus can be applied in individuals with oral language alterations. The examination of aphasic patients indicated that significant differences in relation to a control group occurred in the presentation of stimuli in 2-back conditions.^11^

The performance of normal individuals is important not only to study socio-demographic effects such as age, but also to be used as a parameter to compare normal and brain damaged behavior.

This study aimed to devise material (list of words) to test N-Back working memory in a Brazilian sample and to verify any age effect on performance of the N-Back auditory task.

## Methods

### Participants

Thirty-four adults, members of an elderly group facility seeking to stimulate social, leisure and physical activities, were evaluated.

Thirty-one participants fulfilled inclusion criteria: there was no prior neurologic or psychiatric disease, alcoholism, depressive symptoms or auditory dysfunction. The Mini-mental state examination (MMSE)^12,13^ and Geriatric Depression Scale (GDS)^14^ were used to screen the subjects. Two individuals who presented below cut-off MMSE scores were excluded. One subject with a higher than expected GDS score also was excluded.

Two groups were formed according to age: Group 1 (G1), between 30–60 years and Group 2 (G2), between 60–75 years.

The study was approved by the Ethics Committee – CAPEPesq (Proc n.701/06). The participants signed the Terms of Consent to take part in the study.

### Material

The instrument used was the adapted version of the N-Back auditory,^11^ consisting of 5 items to be processed in WM.

A list of words drawn from different categories was used (foods, animals, man-made things, office objects, furniture, geometric forms, and clothing), and randomly distributed^15^ based on frequency and familiarity according to Brazilian Portuguese criteria.^16^ Syllabic structure (di and tri-syllables) was considered when selecting the target item to be recognized.

Two lists were created to form a total of 60 stimuli. The stimuli were digitally recorded in stereo at 44.1 kHz.

#### Procedures and analysis

The evaluation was performed individually and during a single session within a silent isolated environment at the elderly group facilities. Sessions lasted a maximum of one hour.

**Figure 1:**
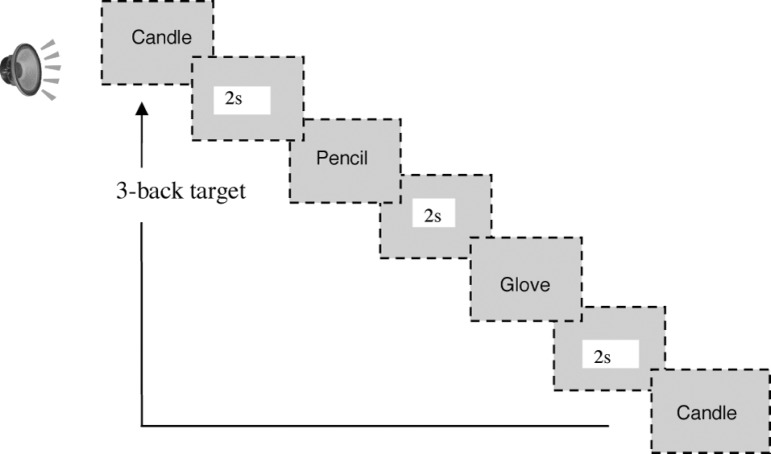
Presentation of items.

The sequences of one-second auditory stimuli, with two second intervals, were presented on binaural headphones. After listening to the sequence, the individuals were shown the items presented, for recognition of the 1-back ... 5-back target (Figure 1).

The subjects were observed by the examiner during the presentation of the sequence, to guarantee their sustained attention. Participants were told to use a gesture to indicate the point at which the stimulus was repeated.

The participant was deemed correct when they appropriately indicated the target-word, and incorrect in the absence of an answer or intrusion of a non-presented item. The intruded items were noted for each sequence. The total number of correct answers and the time span were considered. The latter was scored in accordance with the n of the series. For example, when presenting a 5-item series (5-back), the individual could err on one item in a sequence of 4, and in the last presentation get the target correct in a sequence of five items; in this case, the total score of correct answers would be less than the span, which corresponds to the total of correct answers in the N-Back block.

## Results

The two groups included subjects aged from 3-45 yrs (G1) and 60-74 yrs (G2). The socio-demographic data are presented in Table 1.

**Table 1:** Sociodemographic description*.

Participants	Mean age (SD)	Mean schooling (SD)	Mean GDS (SD)	Mean MMSE (SD)
G1	39.4(4.9)	10.2 (2.8)	6 (1.2)	28.2 (2.4)
G2	63.3 (5.0)	8.6 (3.8)	6.5 (2.1)	27.12 (1.1)
p value	< 0.001	0.20	0.55	0.01

* Student's t Test; MMSE: Mini-Mental State Examination; GDS: Geriatric Depression Scale; SD: standard deviation.

The groups differed on the statistical analysis by age and MMSE. G2 presented lower scores on the MMSE, although values were within the normal range expected for schooling according to Brazilian criteria. There was no difference in years of schooling or depressive symptom scores (GDS).

The performance on the N-Back test is presented in Table 2. There was no intra-group stimulus effect. Individuals from both the G1 and G2 groups processed di and tri-syllables similarly.

**Table 2:** Group and stimuli effect*. Inter and intra-groups comparison on N-Back performance

Stimuli	Parameters of performance G1 × G2											
	Total			p value	Span			p value	Intrusions			p value
	G1	G2			G1	G2			G1	G2		
Dissyllables	4.6 (0.5)	4.4 (0.8)		0.67	4.9 (0.2)	4.5 (0.8)		0.22	0.8 (1.0)	0.5 (0.6)		0.45
Trissyllables	4.8 (0.41)	4.5 (0.6)		0.37	4.8 (0.41)	4.6 (0.6)		0.37	0.7 (0.96)	0.6 (0.9)		0.70
p value*	0.35	0.67			0.53	0.70			0.75	1.0		

* Mann-Whitney test.

There was no difference between groups on any N-Back parameters: total score, span, number of intrusions, for either di or tri-syllable presentations. No age effect was found on this task.

## Discussion

The N-Back test requires storage-plus processing operations. Series of single items are presented. For each item the participant must decide whether it matches what had appeared two items back in the series. The task also involves rehearsal in that the participant must rehearse the set of items held in memory. Successful performance in the task further requires that the participant drop items that are no-longer relevant from memory (e.g. the item that is three items back), add new items as each is presented, and assign the proper back tag to the items in memory. This task involves more than recognition and, according to well documented studies, minor variations of a “storage-only” task can recruit executive processing cerebral regions, typically sensitive to aging decline.^17,18^

Our expectation was to find an ageing effect but no effect was observed. This result should be discussed considering the following points.

Firstly, the adopted N-Back procedure consisted of a string of words, a different set of digits or letters, in which meaning was not present. A study conducted by Van Gerven et al.^19^ found significant differences between older and younger participants on a two digit N-Back task. It is possible that our subjects used a semantic strategy to achieve string storage and attention focus to obtain the correct answer.

Considering that a 5-item span is the minimum capacity expected to be stored in short-term memory, we presented strings containing 5 elements, a number larger than other studies.^4^ Even under this load condition, meaning could have compensated for the memory load.

A second point is that decline due to aging did not compromise performance on the WM in “all” older people. The aging process is characteristically heterogeneous. Some elders are equal to the young while a sub-group is even better than the young at cognitive tasks. Our sample represented a sub-group which matched the young group for performance.

A third point is that our sample did not include elderly older than 75-years, but constituted younger-older subjects. It is plausible to consider that at this age limit, preservation of cognitive capacities will prevail over decline.

A fourth reason is that our elderly subjects did not have language comprehension complaints or other difficulties in everyday-living. This ecological argument of their integrity should not be ignored.

Finally, although the auditory presentation (phonological) could have posed an additional difficulty, since ageing encompasses a number of difficulties in auditory processing, many studies reviewed by Reuter-Lorenz and Jonides have indicated that spatial and not phonological WM were compromised in older groups. Our results are in agreement with these studies.^20^

The N-Back task is a valuable WM measure as it reflects ecological abilities such as comprehension of sentences. We envisage several avenues for future N-Back research, for instance, by increasing the number of participants and including language activities that demand monitoring and executive function, verifying the semantic effect of stimulus in accuracy of answer and span; verifying intrusion effects, reaction time in identifying target items. If the absence of an age effect is consistently verified in larger samples then the task could be useful for cognitive diagnostic purposes.

The age effect on the N-Back task is not always present in elderly subjects. Even with increased back-phonological-demand a number of elderly can successfully complete the task.
